# Genomic Microbial Epidemiology Is Needed to Comprehend the Global Problem of Antibiotic Resistance and to Improve Pathogen Diagnosis

**DOI:** 10.3389/fmicb.2016.00843

**Published:** 2016-06-15

**Authors:** Ethan R. Wyrsch, Piklu Roy Chowdhury, Toni A. Chapman, Ian G. Charles, Jeffrey M. Hammond, Steven P. Djordjevic

**Affiliations:** ^1^The ithree Institute, University of Technology Sydney, SydneyNSW, Australia; ^2^NSW Department of Primary Industries, Elizabeth Macarthur Agricultural Institute, SydneyNSW, Australia; ^3^Institute of Food Research, Norwich Research ParkNorwich, UK

**Keywords:** multiple antibiotic resistance, animal production, environmental pollutants, genomic epidemiology, agriculture, *Escherichia coli*

## Abstract

Contamination of waste effluent from hospitals and intensive food animal production with antimicrobial residues is an immense global problem. Antimicrobial residues exert selection pressures that influence the acquisition of antimicrobial resistance and virulence genes in diverse microbial populations. Despite these concerns there is only a limited understanding of how antimicrobial residues contribute to the global problem of antimicrobial resistance. Furthermore, rapid detection of emerging bacterial pathogens and strains with resistance to more than one antibiotic class remains a challenge. A comprehensive, sequence-based genomic epidemiological surveillance model that captures essential microbial metadata is needed, both to improve surveillance for antimicrobial resistance and to monitor pathogen evolution. *Escherichia coli* is an important pathogen causing both intestinal [intestinal pathogenic *E. coli* (IPEC)] and extraintestinal [extraintestinal pathogenic *E. coli* (ExPEC)] disease in humans and food animals. ExPEC are the most frequently isolated Gram negative pathogen affecting human health, linked to food production practices and are often resistant to multiple antibiotics. Cattle are a known reservoir of IPEC but they are not recognized as a source of ExPEC that impact human or animal health. In contrast, poultry are a recognized source of multiple antibiotic resistant ExPEC, while swine have received comparatively less attention in this regard. Here, we review what is known about ExPEC in swine and how pig production contributes to the problem of antibiotic resistance.

## Antimicrobial Resistance in Agricultural and Clinical Settings

Meat production farming practices have increased in scale over the past 30 years (Landers et al., 2012) and are predicted to continue to increase in line with rising incomes in low- and middle-income countries (Van Boeckel et al., 2015). This rationalization of farming has led to intensification of animal production and greater reliance on antimicrobials to control infectious disease, improve feed conversion efficiency, and promote animal growth (Levy, 1992; Sarmah et al., 2006; FAO, 2009; Krishnasamy et al., 2015; Van Boeckel et al., 2015). Modeling studies estimate that global antimicrobial consumption will increase by 67% from an estimated 63,151 tons in 2010 to 105,596 tons in 2030 (Van Boeckel et al., 2015). Much of this rise in consumption is expected to come from China, India, Russia, South Africa, and Brazil; countries where meat consumption is growing due to rising incomes (Van Boeckel et al., 2015). Large scale animal production facilities generate huge volumes of animal waste that is contaminated with veterinary antibiotics. While estimates vary, it is thought that in the USA, 11–14 million kilograms of antibiotics are used annually in the production of animals for food (i.e., livestock; Institute of Medicine; National Research Council; Panel on Animal Health, 1999; Landers et al., 2012). Almost half of these are used for non-therapeutic purposes, which is significantly more than the estimated 1.4 million kilograms of antibiotics used in human medicine (Institute of Medicine; National Research Council; Panel on Animal Health, 1999). Many classes of antibiotics are used in animal production including β-lactams, sulfonamides, tetracyclines, streptogramins, macrolides, lincosamides, polyethers, quinoxalines, elfamycins, glycolipids, arsenicals, and polypeptides (Sarmah et al., 2006). How these compounds are modified within the animal, how they persist in soils and waterways after they are excreted, and their fate when animal slurries are used to fertilize pastures are complex and poorly understood (Sarmah et al., 2006; Zhu et al., 2013). Antimicrobial residues that contaminate groundwater tables, aquatic environments, and land used for crop production exert selection pressure on microbial populations that may promote the lateral transfer of resistance and virulence genes between populations, and contributes to the emergence of novel pathogenic profiles (Venturini et al., 2010, 2013; Baquero and Tobes, 2013; Baquero et al., 2013; Roy Chowdhury et al., 2015). It is perhaps unsurprising that commensal gastrointestinal bacterial populations are commonly resistant to more than one antimicrobial (Bettelheim et al., 2003; Diarrassouba et al., 2007; Bailey et al., 2010) and consequently the frequency of community-acquired infections with resistance to multiple antimicrobials are also increasing (Onken et al., 2015; Paniagua-Contreras et al., 2015; Yahiaoui et al., 2015). Furthermore, surveys have shown the presence of antibiotic resistance genes in the gastrointestinal tracts of humans and animals that have never received antibiotic interventions, specifically in: human feces from remote communities (Bartoloni et al., 2009; Ruppe et al., 2009; Eisenberg et al., 2012), migratory bird populations (Foti et al., 2011; Carroll et al., 2015; Jamborova et al., 2015; Stedt et al., 2015) and various wildlife species (Allen et al., 2010; Baez et al., 2015; Jobbins and Alexander, 2015; Katakweba et al., 2015). This demonstrates that resistance genes in host bacteria can readily spread to naïve microbial populations via waterways, air currents, road incursions into remote areas, animal and bird migration, and land clearance (Tsubokura et al., 1995; Middleton and Ambrose, 2005; Ewers et al., 2009). The application of manure onto pasture from ruminants that have not received recent antibiotic therapy is known to stimulate the expansion of resident flora encoding resistance to β-lactam antibiotics (Udikovic-Kolic et al., 2014). These observations demonstrate our limited understanding of the impact of the application animal waste to agriculture. The mechanisms by which mobile elements flux through microbial populations are not fully understood, but it is clear that efforts must be made to reduce environmental contamination sourced from human and animal effluent.

Antimicrobial resistance is largely an ecological problem (Summers, 2002, 2006; Stokes and Gillings, 2011; Landers et al., 2012; Berendonk et al., 2015; Gillings et al., 2015). In general, studies have focused on the emergence and carriage of resistance genes in certain key microbial populations, without an appreciation for the wider complexity of the problem, or its origins. Hence, there are numerous under-reported microbial sources of resistance genes. Antibiotic resistance genes often traffic in association with transposons that carry mercury resistance operons; these play an important role in transforming mercury to less biologically toxic forms, thereby regulating the availability of toxic mercury compounds in the environment. Mercury-resistance gene operons have evolved as key components of Tn*3*-family transposons and these transposons are globally disseminated. Mercury has been released into the environment via geological processes over millions of years and, although anthropogenic activity over the past two centuries has contributed significantly to the release of mercury into waterways and the atmosphere, it is likely that mercury levels were higher prior to the industrial revolution than they are now as a result of extensive volcanic activity (Osborn et al., 1997; Barkay et al., 2003). Despite this, large scale surveillance studies of antimicrobial resistance fail to screen microbial populations for the presence of mercury-resistance genes (Djordjevic et al., 2013). Resistance genes can also accumulate in microbial populations in soils that have been fertilized with animal waste and treated effluent (Heuer and Smalla, 2007; Chee-Sanford et al., 2009; Heuer et al., 2011a,b; Kristiansson et al., 2011; Baquero et al., 2013; Berendonk et al., 2015). Meat, seafood, and vegetables are also important and under-recognized sources of antimicrobial resistance genes in bacteria (Johnson et al., 2005a; Agerso et al., 2014; Kim and Woo, 2014; Ahmed and Shimamoto, 2015a,b; Ben Said et al., 2015; Le et al., 2015; van Hoek et al., 2015; Zhang et al., 2015; Zurfluh et al., 2015).

Anthropogenic industrial processes generate heavy metals including cadmium, lead, arsenic, copper, silver, and a wide range of synthetic organic compounds that pollute natural ecosystems. As such, heavy metal contamination represents yet another selective pressure for the development of antimicrobial resistance (Baker-Austin et al., 2006; Castillo et al., 2008; Li et al., 2010; Zhu et al., 2013). Genes coding for resistance to silver, copper, arsenic and antimony are associated with complex resistance gene loci in bacteria resistant to more than one antimicrobial and have great significance to human, livestock, and plant health (Berg et al., 2005; Seiler and Berendonk, 2012; Hobman and Crossman, 2015; Bondarczuk et al., 2016). The widespread occurrence of genes coding for resistance to heavy metals has, in part, been demonstrated in studies using information mined from publicly available genome sequences highlighting the value of genomic surveillance studies (Hobman and Crossman, 2015).

Genome-wide studies of genes coding for resistance to antibiotics, biocides and heavy metals have shed light on how these genes co-assemble on mobile genetic elements. In an analysis of 2522 fully sequenced bacterial genomes and 4582 plasmid sequences, Pal et al. (2015) were able to identify examples where antimicrobial resistance genes co-occurred. The authors found that the most likely co-selection scenario occurred in bacterial strains that carried plasmid-borne antibiotic resistance genes and biocide/heavy metal resistance genes on the same chromosome. A plasmid localized gene cluster coding for a cadmium/zinc resistance gene (*cadD*) with aminoglycoside and macrolide resistance genes was also identified (Pal et al., 2015). Resistance to these combinations of antimicrobials would benefit bacterial populations found in intensive animal production systems where cadmium and zinc are both used to promote growth and aminoglycosides and macrolides are frequently administered for the treatment of Gram-negative and Gram-positive infections, respectively (Castillo et al., 2008; Li et al., 2010). It is clear that plasmids can carry combinations of genes coding for resistance to more than one microbial agent alongside genes coding for resistance to biocides and heavy metals. Invariably these plasmids are large, conjugative and commonly found in pathogens from hospital and intensive farming environments (Chen et al., 2007; Woodford et al., 2009; Venturini et al., 2010, 2013; Pal et al., 2015). Sub-lethal levels of antibacterial biocides and heavy metals found in mildly polluted aquatic and land-based ecosystems may be sufficient to maintain these mobile elements carrying multiple antibiotic, biocide and heavy metal resistance genes (Stepanauskas et al., 2006; Gullberg et al., 2014).

Although plasmids can vary in size and coding ability, their size is generally positively correlated with the size of the host chromosome (Smillie et al., 2010). Plasmids conferring multiple-drug resistance are usually large, as they code for a suite of resistance genes, alongside associated integrons, transposition factors, and genes for heavy metal resistance, which can be in excess of 20,000 bp or more in size. Small plasmids that acquire resistance to more than one antimicrobial are, however, not without precedent. Labar et al. (2012) described the sequence of a small plasmid (pASL01A; 27,072 bp) that codes for resistance to ampicillin, streptomycin, sulfonamides, trimethoprim, and mercury (Labar et al., 2012). Most of the plasmid (21,904 bp) is comprised of a Tn*21* derivate mercury resistance transposon that carries a class 1 integron with a trimethoprim resistance *dfrA7* gene cassette and Tn*6029C* (Roy Chowdhury et al., 2015). Tn*6029C* comprises a *bla*_TEM-1_-IS*26-repA/C-sul2-strA-strB* gene cluster flanked by direct copies of IS*26* (Labar et al., 2012; Roy Chowdhury et al., 2015). The plasmid backbone (5,168 bp) and related plasmids are widespread in enterobacterial populations in Africa (Labar et al., 2012).

Antimicrobials, including heavy metals, are used widely in animal production to treat clinical disease, to prevent disease outbreaks during critical or vulnerable periods, and to promote growth (McEwen and Fedorka-Cray, 2002; Landers et al., 2012). The use of antibiotics to promote growth is a phenomenon whereby animals receiving very low doses of antimicrobials appear healthier and grow larger (Roy Chowdhury et al., 2014). Although, the mechanism for this growth promoting effect is still poorly understood, the majority of pigs produced in the USA are exposed to tetracyclines and tylosin to prevent disease and promote growth (Apley et al., 2012). While these approaches have proved effective in controlling disease and aiding farms globally, the uninhibited use of antimicrobials also provides the selection pressure that drives antimicrobial resistance (Pulcini and Gyssens, 2013). As such, concerted efforts to reduce the use of antimicrobials in food production should be a priority.

Given that the persistence and spread of resistance is intrinsically linked with the presence and activity of antimicrobials, it is significant that 30–90% of ingested antibiotics are excreted in an un-metabolized or only partially metabolized form (Sarmah et al., 2006) into waste treatment plants and animal waste-holding facilities. Antibiotic residues are known to persist in secondary effluent despite the treatment process (Sarmah et al., 2006; Watkinson et al., 2007; Martinez, 2008; Le-Minh et al., 2010; Bondarczuk et al., 2016). Furthermore, the pH, temperature, nutrient concentration, and bacterial loads in waste treatment plants are conducive to the evolution and spread of antimicrobial resistance (and virulence) genes (Andersen, 1993; Dolejska et al., 2011). This is in part evidenced by an increase in the presence of the class 1 integrase gene, a reliable proxy for resistance to more than one antimicrobial in bacterial populations sampled in wastewater treatment plants (LaPara et al., 2011; Ma et al., 2013; Gillings et al., 2015), and an increase in the number of resistance genes in wastewater treatment plants and rural domestic wastewater treatment systems (Gao et al., 2012; Chen and Zhang, 2013; Ju et al., 2015; Mao et al., 2015). This indicates that controlling the use of antimicrobials is a prerequisite for slowing the development and spread of pathogens with multiple antimicrobial resistance mechanisms.

## Antimicrobial Resistance in *Escherichia coli*

*Escherichia coli* is a widespread and abundant organism capable of causing a wide range of gastrointestinal and extra-intestinal diseases. It can survive and proliferate in a diversity of terrestrial and aquatic environments (van Elsas et al., 2011). As such, its genetic profile is shaped profoundly by horizontal gene transfer, and it is a useful marker species for understanding how antimicrobial resistance and virulence genes accumulate over evolutionary time. Horizontal gene transfer events are known to generate novel combinations of putative and established virulence factors, and generate novel pathotypes of *E. coli* (Wu et al., 2007; Bielaszewska et al., 2014). Although, *E. coli* is a commensal inhabitant of the gastrointestinal tract of warm-blooded animals, pathotypes have evolved that cause intestinal [intestinal pathogenic *E. coli* (IPEC)] and extra-intestinal [extraintestinal pathogenic *E. coli* (ExPEC)] diseases. Different pathotypes of *E. coli* cause diarrheal disease, hemolytic uremic syndrome (HUS), urinary tract infection (UTI), pyelonephritis, septicemia, meningitis, and respiratory disease (pneumonia) in humans and animals. Known IPEC pathotypes include the attaching and effacing *E. coli* (AEEC), Shiga-toxigenic *E. coli* (STEC), enteroaggregative *E. coli* (EAEC), enterotoxigenic *E. coli* (ETEC), enteroinvasive *E. coli* (EIEC), diffuse adhering *E. coli* (DAEC) and the recently described enteroaggregative hemorrhagic *E. coli* (EAHEC; Kaper et al., 2004; Brzuszkiewicz et al., 2011). EHEC are a subgroup of STEC, which colonizes the terminal regions of the gastrointestinal tract of ruminants. It is notable that different serotypes of *E. coli* preferentially colonize different ruminant hosts (Djordjevic et al., 2001, 2004; Hornitzky et al., 2002) and carry different Shiga toxin gene variants (Ramachandran et al., 2001; Brett et al., 2003a,b). These studies underscore the importance of understanding the ecology of disease-causing organisms and how niche adaptation influences lateral gene transfer. Notably, however, ruminants are not a recognized source of ExPEC (Manges and Johnson, 2015).

Many recent molecular surveillance studies on *E. coli* report the frequency of genes that code for extended-spectrum beta lactamase enzymes (ESBL’s), which are indicative of resistance to penicillin, cephalosporin, and other clinically important antibiotics, but fail to screen for resistance to the older, first generation antibiotics (Bean et al., 2009; Ewers et al., 2012; Glasner et al., 2013; Roschanski et al., 2015). Genes that code for resistance to first generation antibiotics are often carried in bacteria containing class 1 integrons (Johnson et al., 2005b; Jakobsen et al., 2011; Hansen et al., 2014). From a diagnostic perspective, recent genomic studies on an outbreak of EAHEC and EAEC strain O104:H4, highlighted this issue (Ahmed et al., 2012). EAHEC is a recently emerged *E. coli* pathotype and EAEC strain O104:H4 has acquired the Shiga toxin gene *stx2a*, a diagnostic marker of EHEC. Outbreak strains were found to be resistant to ampicillin, streptomycin, sulfamethoxazole, trimethoprim, and tetracycline but little effort was given to determining how the genes that coded for these antibiotic resistance phenotypes were assembled (Ahmed et al., 2012). We were subsequently able to show that the genes coding for resistance to these antibiotics were assembled in Tn*6029D*, a transposon that abuts a class 1 integron in a Tn*21*/Tn*1721* hybrid backbone (Roy Chowdhury et al., 2015). The resistance region resided in genomic island 3 which also carried the virulence gene *ag43* encoding a self-associating serine protease auto-transporter, important for biofilm formation. This is an example of how antimicrobial resistance genes and virulence genes co-localize on the same mobile genetic element (Roy Chowdhury et al., 2015). Chromosomal islands are increasingly associated with carriage of class 1 integrons encoding resistance to multiple antibiotics of clinical significance (Roy Chowdhury et al., 2016).

Salmonella genomic island 1 (Boyd et al., 2001; Levings et al., 2005), Salmonella genomic island 2 (Levings et al., 2008) and several large plasmids in EHEC strain O26:H (Venturini et al., 2010), and an atypical EPEC, strain O111 (Venturini et al., 2013) are also examples of virulence and resistance genes being carried on the same mobile element. Strains of *E. coli* that carry genes that are markers for more than one *E. coli* pathotype pose a dilemma for diagnostic laboratories. There are numerous reports describing novel *E. coli* strains with combinations of virulence genes that are diagnostic of more than one *E. coli* pathotype; this suggests that newly emerging strains are increasingly likely to have complex resistance loci facilitated by the clustering of mobile element-associated resistance and virulence genes. In a study of 265 cases of *E. coli* causing UTI, 28 carried virulence genes typical of IPEC (Bielaszewska et al., 2014). Several other examples of hybrid *E. coli* strains have been described and their presence demonstrates the need to have genomic surveillance strategies in place to determine how antibiotic resistance and virulence genes assemble in emerging pathogens (Wallace-Gadsden et al., 2007; Abe et al., 2008; Bielaszewska et al., 2014; Mariani-Kurkdjian et al., 2014).

Extraintestinal pathogenic *E. coli* is a phylogenetically diverse group comprised of a broad range of *E. coli* sequence types (STs). ExPEC are fecal *E. coli* that rarely cause intestinal disease but have acquired a wide variety of virulence gene cargo that facilities their ability to cause disease at extraintestinal sites, particularly in the urinary tract. ExPEC are the most frequently isolated Gram negative bacterial pathogen infecting humans incurring significant mortality and morbidity. Notably, ExPEC are a leading cause of sepsis and are increasingly resistant to multiple antibiotics posing a serious health concern (Manges and Johnson, 2015; Poolman and Wacker, 2016). ExPEC have a fecal origin but rarely cause gastrointestinal disease. The acquisition of a diverse range of virulence factors by lateral gene transfer has armed ExPEC with an ability to cause life-threatening blood-borne, urinary tract, respiratory, skin and soft tissue infections, and meningitis in humans of all ages (Russo and Johnson, 2003; Poolman and Wacker, 2016). ExPEC vary widely in the combinations of putative virulence genes that they carry. Many genes encode for adhesins, iron acquisition systems and other putative virulence attributes and this genetic redundancy complicates efforts to develop broadly applicable molecular diagnostic tests to detect ExPEC (Johnson et al., 2001; Spurbeck et al., 2012). Importantly, livestock are important reservoirs for ExPEC (Ewers et al., 2007, 2009; Manges et al., 2007; Vincent et al., 2010; Nordstrom et al., 2013; Manges and Johnson, 2015). Virulence genes are often shared among *E. coli* isolates from samples collected from humans, and the meat and feces of intensively reared livestock, particularly poultry (Rodriguez-Siek et al., 2005; Vandekerchove et al., 2005; Jakobsen et al., 2010a,b). Consistent with this view, ExPEC carry genes coding for resistance to antimicrobials frequently used in veterinary medicine. Hospital diagnostics laboratories do not screen for most of the antibiotics used in veterinary medicine and so resistance to these is under-reported. In a Chinese study of 315 ExPEC isolates from various pig tissues, more than 63% exhibited resistance to 10 antibiotics including ampicillin, trimethoprim, sulfadimidine, tetracycline, neomycin, streptomycin, and kanamycin (Tan et al., 2012). The genes *bla*_TEM-1_ (ampicillin resistance); *strAB* (streptomycin resistance); *sul1* and, *sul2* (sulfonamide resistance); *aphA1* (kanamycin and neomycin resistance); *tetA*, *tetB*, and *tetG* (tetracycline resistance); as well as various *dfrA* genes (trimethoprim resistance) coded for resistance to these antibiotics.

**FIGURE 1:**
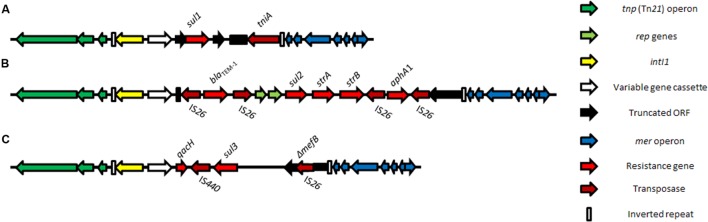
**Class 1 integron structures associated with the three sulfonamide resistance gene alleles *sul1***(A)**, *sul2***(B)**, and *sul3***(C)**, shown in a *Tn21* backbone**.

The class 1 integron is widespread in clinical settings. Bacteria that are resistant to more than one antimicrobial often carry class 1 integrons and their presence is a reliable indicator of multiple antimicrobial resistance (Leverstein-van Hall et al., 2003). The class 1 integron structures identified in most clinical samples are thought to be derived from the capture of a chromosomally located class 1 integron, prevalent within environmentally abundant bacterial species belonging to the broad β-proteobacterium group, by a Tn*402* family transposon (Gillings et al., 2008; Gillings, 2014). Global spread of the class 1 integron is in part a result of the propensity for the Tn*402* family of transposons to target *res* sites found on a wide variety of conjugative and mobile plasmids, including the widespread mercury-resistance transposon, Tn*21*, and the broader Tn*3* family (Liebert et al., 1999; Gillings et al., 2008, 2015). For this and a variety of other reasons described below, resistance genes cluster on mobile elements. Clinical class 1 integrons have subsequently evolved, typically by the loss of most of the Tn*402 tniB* transposition gene, and partial fusion of the *qacE* gene (coding for an efflux pump for disinfectants, Hall et al., 1994; Partridge et al., 2001) with the sulfonamide resistance gene *sul1*. The most commonly observed structure observed in clinical isolates (**Figure 1A**) is comprised of a 5′ conserved segment (5′-CS), a 3′-conserved segment (3′CS) and a variable region inserted in between them. The 5′-CS contains the *intI1* gene encoding the class 1 integrase; the Tn*402* inverted repeat, the two promoters, one required for the transcription of the integrase and the other for the gene cassettes and the *attI1* recombination site. The variable region contains a diverse array of resistance gene cassettes (Partridge et al., 2009) and the associated *attC* sites. The 3′ conserved segment (3′-CS) most frequently contains *qacEΔ1* and *sul1* genes, remnants of the transposition module and a terminal Tn*402* inverted repeat. Most gene cassettes found in clinical class 1 integrons from nosocomial and livestock settings code for resistance to trimethoprim (*dfrA*) and aminoglycosides (*aadA*); despite this over 130 different gene cassettes have been described (Partridge et al., 2009) and, to date, 410 annotated variants are recognized (Tsafnat et al., 2011).

Further variation in the structure of the 3′-CS has also been described (Pan et al., 2006; Cain et al., 2010; Dawes et al., 2010; Saenz et al., 2010; Venturini et al., 2010, 2013; Mattiello et al., 2015). In one instance, the 3′-CS was almost completely lost and had been replaced with the compound transposon, Tn*6026* (Venturini et al., 2013). The Tn*6026* structure contains the beta-lactamase *bla*_TEM-1_ gene; the sulfonamide resistance gene, *sul2*; the streptomycin resistance gene *strAB*; the neomycin/kanamycin resistance gene, *aphA1* and it is flanked by direct copies of IS*26* (**Figure 1B**). Tn*6026* is located between *attI*, the insertion point of the gene cassette, and the defective *tni* module of a clinical class 1 integron, replacing all but the first 24 bp of the 3′-CS (Cain et al., 2010; Dawes et al., 2010; Venturini et al., 2010, 2013). A *sul3* variant structure associated with IS*26* has also emerged over the last decade from human- and animal-sourced isolates (**Figure 1C**). The first report of *sul3* was from Swiss pigs and humans in Grape et al. (2003) and Perreten and Boerlin (2003). Since then, *sul3* has been reported in samples from a number of countries (Pan et al., 2006; Saenz et al., 2010; Tang et al., 2011; Mattiello et al., 2015; Wyrsch et al., 2015; Moran et al., 2016). These atypical class 1 integrons (**Figure 1**) are often components of larger complex resistance regions, hosted on plasmids that also carry virulence genes (Venturini et al., 2010, 2013).

Small mobile elements, including insertion sequences (IS*1*, IS*26*, IS*Ecp1c*, and IS*6100* are of note), Insertion Sequence Common Regions (ISCRs) and composite transposons formed from two or more IS elements, play an important role in reshaping the 3′-CS of clinical class 1 integrons (Daly et al., 2005; Dionisi et al., 2009; Venturini et al., 2010). The genes *blaSHV-11*, and *blaSHV-12* (coding for β-lactam resistance; Ford and Avison, 2004; Dionisi et al., 2009); *qnrB19* (coding for quinolone resistance; Dionisi et al., 2009); and *aphA1* (coding for kanamycin/neomycin resistance; Cain et al., 2010) have been shown to be flanked by direct copies of IS*26*. The mechanism of action of IS*26* has been the subject of several recent studies (Harmer et al., 2014; Harmer and Hall, 2015; He et al., 2015). These studies show that resistance loci containing IS*26* can be hotspots for the capture of further resistance genes and other genetic cargo flanked by IS*26* and demonstrates the important role of IS*26* and other mobile elements in the assembly and re-assembly of novel complex resistance loci.

Currently, genomic epidemiological surveillance relies heavily on whole genome and metagenomic sequence information generated using short-read (<400 bp) sequencing platforms. A fall in the cost of high throughput, next generation sequencing with platforms that generate short-read lengths means they have become the leading technology for characterizing microbial evolution and uncultured microbial consortia, and to catalog antimicrobial resistance and virulence gene carriage. However, short-read sequencing technologies are unable to assemble complex resistance gene loci, or generate complete genome sequences, without which it is difficult to characterize mobile genetic elements rapidly and reliably. These limitations severely restrict efforts to comprehend the role of lateral gene transfer in the evolution of microbial pathogens and antimicrobial resistance, particularly in regards to the microevolutionary events that lead to the generation of novel complex resistance loci. Long-read sequencing technologies have improved significantly over the past 5 years and are beginning to address this shortfall, but are currently either cost prohibitive for large-scale genomic epidemiological surveillance or require further improvements in base call accuracy (Loman and Pallen, 2015).

Genes that code for resistance to older-generation antibiotics are often incorporated within complex resistance regions. In part, this provides a plausible explanation for why these genes persist despite enforced restrictions on prescribing of older-generation antibiotics. In the UK, strict limitations on the prescription of sulfonamide drugs were enforced in Anonymous (1995). However, functional *sul* genes persist in clinically important Gram negative bacteria and their frequency has not declined since the restriction was imposed (Enne et al., 2001; Bean et al., 2005, 2009). A similar scenario unfolded in Finland when trimethoprim was withdrawn from use for the treatment of UTI (Heikkila et al., 1990). In the Enterobacteriaceae, resistance to sulfonamides, trimethoprim, ampicillin, tetracycline, and fluoroquinolones remains high (Enne et al., 2001; Nozarian and Abdollahi, 2015; Paniagua-Contreras et al., 2015; Uzun et al., 2015; Yahiaoui et al., 2015). Carriage rates for *sul1*, *sul2*, or *sul3* genes in *E. coli* isolated from retail meat, livestock, healthy humans, and humans with clinical disease also remains a problem (Enne et al., 2001; Blahna et al., 2006; Frank et al., 2007; Altalhi et al., 2010; Bailey et al., 2010; Saenz et al., 2010; Vogt et al., 2014; Yahiaoui et al., 2015). Carriage of *sul* genes is linked with the presence of both typical and atypical class 1 integrons, including genes that code for resistance to ampicillin, streptomycin, and trimethoprim (Bettelheim et al., 2003; Bean et al., 2009; Bailey et al., 2010; Soufi et al., 2011; Ewers et al., 2014; Yahiaoui et al., 2015). Combinations of genes coding for resistance to first generation antibiotics contribute significantly to the multiple antimicrobial resistance phenotypes observed in commensal *E. coli* (Bettelheim et al., 2003; Vinue et al., 2008, 2010; Bailey et al., 2010). Genetic studies that have examined resistance to a broad spectrum of antibiotics frequently report the presence of *bla*_TEM-1_, *sul1*, *sul2*, *strAB*, *tetA*(A), and a range of *aadA* and *dfrA* genes (Soufi et al., 2011; Labar et al., 2012; Ewers et al., 2014; Yamamoto et al., 2014; Yahiaoui et al., 2015). The streptomycin resistance gene, *strAB*, and the *sul2* sulfonamide resistance gene are also spread via small plasmids such as RSF1010 (Yau et al., 2010), and IncQ plasmids that are related to RSF1010 (Tietze et al., 1989). The *strAB* gene and the adjacent inverted repeat IR_R_, as seen in RSF1010, probably had their origins in Tn*5393c*, while *sul2* was recruited from a CR2-containing element (Yau et al., 2010). These molecular events describing the evolution of the *sul2-strAB* configuration observed in RSF1010 have been described previously (Yau et al., 2010). The *sul2-strAB* gene cluster and flanking sequences from RSF1010 have been captured onto Tn*21* derivative transposons, on large and small plasmid backbones, and in chromosomal islands; they are often in association with IS*26* and are distributed widely (Daly et al., 2005; Cain et al., 2010; Venturini et al., 2010, 2013; Labar et al., 2012; Reid et al., 2015; Roy Chowdhury et al., 2015).

## *E. coli* in Intensive Production Animals

### Porcine Enterotoxigenic *Escherichia coli* (ETEC)

Enterotoxigenic *Escherichia coli* (ETEC) are the causative agents of three distinct diseases in young pigs: neonatal, pre-weaning, and post-weaning diarrhea (Fairbrother et al., 1988; Noamani et al., 2003). These diseases vary in severity and are categorized by the age at which the pig develops symptoms. Diarrhea caused by ETEC strains, generally known as enteric colibacillosis, is a problem in all countries involved in pig production, and various methods have been employed to protect pigs from ETEC infection and any subsequent diarrheal disease. These methods include, but are not limited to, treatment with antibiotics, vaccination, and feed modulation (Fairbrother et al., 2005; Virdi et al., 2013). While these protective steps have helped reduce levels of ETEC infection, particularly in neonatal and pre-weaning colibacillosis, antimicrobial resistance has become a global challenge that is severely limiting antimicrobials as a treatment option.

Pathogenic *E. coli* express a wide variety of functional molecules including toxins, adhesins, autotransporters, and invasins (Nataro and Kaper, 1998; Kaper et al., 2004), which enable the *E. coli* cells to damage host tissues. In porcine ETEC, these molecules, referred to as virulence factors, include heat-stable (ST) and heat-labile (LT) enterotoxins, both of which can cause diarrhea via different molecular pathways within the pig’s gastrointestinal tract (Nataro and Kaper, 1998). Various adhesins have evolved in ETEC and are expressed on the bacterial cell surface, allowing adhesion to the host’s gastrointestinal tract. In pigs, the K88 (F4), K99 (F5), 987P (F6), F18 and F41 fimbrial adhesins all provide host intestinal cell binding, with K88, K99 and F18 fimbriae being particularly prevalent (Nataro and Kaper, 1998; Nagy and Fekete, 1999; Koh et al., 2008). Interestingly, the K88 and F18 fimbrial adhesins are often associated with pre- and post-weaning diarrhea, whilst the K99 fimbria has been associated with neonatal diarrhea in pigs (DebRoy and Maddox, 2001). These binding effects are thought to be supplemented by other common adhesins coded by genes such as *fimH* (Bouckaert et al., 2006) and *eaeH* (Sheikh et al., 2014), which are known to provide additional post-attachment binding. Chapman et al. (2006) employed multiplex PCR assays targeting 58 virulence genes to screen 52 clinical *E. coli* isolates from episodes of porcine neonatal and post-weaning diarrhea, and 23 commensal *E. coli* isolates from healthy pigs. Of the 58 genes, 17 were useful in distinguishing commensal from clinical *E. coli*, nine of which (*iha*, *hlyA*, *aidA*, *east1*, *aah*, *fimH*, *iroN_E.coli_*, *traT*, and *saa*) were identified for the first time in clinical porcine isolates in Australia (Chapman et al., 2006).

In an attempt to combat *E. coli* expressing these and other virulence genes, antimicrobials have been employed as both curative and prophylactic medicines on farms worldwide. This has placed a selection pressure on both targeted and surrounding microbial populations, and as such resistance to these antimicrobials has become extremely prevalent. For pathogenic *E. coli* in general, the definition and interpretation of virulence has been tempered based on results from large-scale microbial genome sequencing projects (Falkow, 1988; Pallen and Wren, 2007). ExPEC reside in the gut of their host, often in a commensal state, but have the genetic repertoire of virulence genes that enables them to cause life-threatening extraintestinal infections. Due to the degree of redundancy inherent in many ExPEC virulence genes, it remains a challenge to define virulence in ExPEC. These ExPEC-associated virulence genes have also been found in isolates sourced from humans, poultry and swine but rarely in cattle and other ruminants (Johnson et al., 2001; Tan et al., 2012; Manges and Johnson, 2015). Poultry are a well-recognized reservoir of ExPEC that have the potential to cause human disease but the role of swine in this regard has not been investigated sufficiently.

### Current Status of Global Antimicrobial Use and Its Influence on the Porcine *E. coli* Resistome

Rationalization of farming practices has had profound effects on the spread of resistance to antimicrobials, and consequently both human and animal health – and the problem continues to grow (FAO, 2009). An estimated 210 million kilograms of antibiotics are produced annually in China, making it the largest producer and consumer of antibiotics globally. Approximately half of the antibiotics produced are used in livestock production (Hvistendahl, 2012; Zhu et al., 2013). China is also the largest producer and consumer of pork; 690 million pigs were produced in 2011 and 38.1 kg of pork are consumed per person per year (Krishnasamy et al., 2015), now reportedly almost double the pork consumption of the USA population. China is said to contain half the world’s pig population, with five times the production rate of the USA (the former largest production center) and a higher per capita meat consumption than any developing nation other than Brazil and Latin America (FAO, 2009). Trends are similar for poultry production in China (Krishnasamy et al., 2015). An estimated 34 million kilograms of antibiotics, particularly tetracyclines, sulfonamides, macrolides, and penicillins were used in Chinese pig production during 2012 (Krishnasamy et al., 2015). Combination therapies of chlortetracycline with sulfathiazole and penicillin, and chlortetracycline with sulfamethazine and penicillin, are used widely. Other individual antibiotics that are used less extensively include tylosin, chlortetracycline, oxytetracycline, bacitracin, and bambermycin amongst others (Krishnasamy et al., 2015). Furthermore, pig production in China is responsible for the production of approximately 618 billion kilograms of manure per annum (Wang et al., 2006), much of which is contaminated with antibiotic residues and heavy metals, particularly tetracyclines, sulfonamides and copper; some residues are present at levels in the order of 100s of milligrams per kilogram (Pan et al., 2011; Qiao et al., 2012; Zhu et al., 2013). Elevated levels of zinc and arsenic have also been detected in these studies. Of 149 unique antimicrobial resistance genes 63 were detected significantly more frequently in microbes from manure at different stages of processing on three large pig production farms in China, compared with microbes in manure from control animals that received no antimicrobials; this included genes coding for resistance to antibiotics used to treat humans (Zhu et al., 2013). Samples of pig manure from China were also rich in microbes carrying transposase genes, notably those belonging to the IS*6*-family, predominantly IS*26* transposase (Zhu et al., 2013).

In the USA, estimates suggest that the usage of tetracyclines is as high as in China, both in the combinations described above, and as stand-alone treatments. Additionally, bacitracin, carbadox, lincomycin, neomycin, penicillin, tiamulin, tilmicosin, tylosin, and viginiamycin have all been used in the USA pig production industry (Apley et al., 2012). The USA and China combined produce the majority of the world’s pigs through intensive farming practices (FAO, 2009), with China producing up to five times more than the USA (Krishnasamy et al., 2015). However, other countries also have pig production systems that rely heavily on antibiotic usage. In Alberta, Canada, the same suite of antibiotics is used as in the USA, with the addition of dimetridazole (Rajic et al., 2006). A similar pattern of use has also emerged in an Australian survey of pig production, with the use of apramycin and neomycin, penicillins, macrolides, sulfonamides, tetracyclines, lincomycin and spectinomycin, tiamulin, olaquindox, ceftiofur, and dimetridazole reported (Jordan et al., 2009). A number of these antibiotics, including lincomycin, spectinomycin, and dimetridazole are also used in the treatment of infectious human diseases. In an Australia-wide study of 114 porcine-derived *E. coli*, resistance to numerous antibiotics was common: tetracycline (88.6%), ampicillin (71.05%), trimethoprim/sulfamethoxazole (67.5%), streptomycin (69.3%), chloramphenicol (44.74%), neomycin (35.96%), apramycin (34.21%), gentamicin (28.95%), florphenicol (26.32), cefalotin (24.56%), and spectinomycin (21.93%). Resistance to imipenem and amikacin was not detected (Abraham et al., 2015). Of the 114 isolates evaluated 79% were classified as resistant to antibiotics from three or more different classes. Resistance to extended-spectrum cephalosporins (3%) and fluoroquinolones (1%) was detected in isolates belonging to some *E. coli* lineages (ST117, ST744, ST10, and ST1) albeit infrequently (Abraham et al., 2015).

Colistin is a polymyxin antimicrobial used to treat extensively drug-resistant Gram negative infections. It was first used in the 1950s but its use in humans has declined due to concerns about nephrotoxicity and neurotoxicity (Nation and Li, 2009). China, India, and Europe still use colistin extensively for agricultural purposes. In Europe, colistin is used to treat enterobacterial infections in pigs (neonatal diarrhea), poultry (colibacillosis), cattle (neonatal diarrhea in veal calves), sheep, and goats (Timmerman et al., 2006; Pardon et al., 2012); in 2010 it was the fifth most commonly sold antimicrobial after tetracycline, penicillins, sulfonamides, and macrolides (European Medicines Agency [EMA], 2013). Colistin is also used in aquaculture (Xu et al., 2012). The recent emergence of colistin resistance in commensal porcine *E. coli* isolates in China via the acquisition of plasmid-mediated *mcr-1* gene has serious implications for the treatment of pan-drug-resistant Gram-negative bacteria, particularly isolates of the extremely drug resistant *Acinetobacter baumannii* and carbapenemase-resistant *Klebsiella pneumoniae* (Liu et al., 2015). The seriousness of this finding is clear due to: (i) high *in vitro* transfer rates among *E. coli* by conjugation and the ability of the *mcr-1* plasmid to transfer and be maintained in globally dispersed pathogenic clones such as *E. coli* ST131, *K. pneumoniae* ST11, and *Pseudomonas aeruginosa*; (ii) the high frequency of carriage of *mcr-1* in *E. coli* from livestock and in retail meats sourced from southern China; (iii) detection of *mcr-1*-like genes in Malaysia (Liu et al., 2016). The apparent high rates of carriage of *mcr-1* in isolates of *E. coli* from animals and the apparent low incidence in human-derived *E. coli* populations has led to speculation that the heavy use of colistin in agriculture in China has been the main driver for the emergence of plasmid-mediated colistin resistance (European Medicines Agency [EMA], 2013; Liu et al., 2016).

Genes conferring antimicrobial resistance have been identified in *E. coli* from diverse pig-related sources (**Table 1**). The genes *bla*_TEM_, *sul1/2/3*, *aadA*, *dfrA*, and *tetA/B/C* feature prominently. In order, these genes confer resistance to penicillin, sulfonamides (such as sulphamethoxazole), streptomycin and spectinomycin, trimethoprim, and tetracyclines; all of these are used in the treatment of animals. Additional clinically important cases of resistance are also identified sporadically in the literature, specifically to chloramphenicol (*cmlA, cat*), streptothricin (*estX/sat*), florfenicol (*flo*), quinolones (*qnr*), streptomycin (*strAB*), and fosfomycin (*fosA*). There are also a number of known resistance mutations in the *gyr* and *pmr* genes that confer elevated resistance to quinolones and colistin respectively. Already the presence of these genes together in the same isolate and from disparate countries indicates they are both genetically linked in antimicrobial resistance-encoding loci, and globally disseminated. Detection of the genes *bla*_OXA_, *bla*_SHV,_ and *bla*_CTX-M_ coding for the extended-spectrum beta-lactamases, which are relevant to human medicine, is also of concern (Randall et al., 2014). Of note, these data were collected from a variety of publications for each country, and show some selection bias concerning the genes detected. Whole genome sequencing has become a tool to rectify this bias, and will continue to feature in the detection and characterisation of antimicrobial resistance, its evolution and dissemination (Salipante et al., 2015).

**Table 1:** Molecular characterisation of published antimicrobial resistant, porcine-derived *Escherichia coli* isolates.

Country	Resistance genes detected			Phenotypic resistances tested?	Molecular typing	Prominent Achtman MLSTs	Plasmid typing	Virulence gene detection?	Reference
		
	Integron associated	Resistance	Beta-lactamases						
Argentina	*intI2*			Yes	Serogrouping			Yes	Moredo et al., 2007, 2015; Pantozzi et al., 2010
Australia	*intI1, intI2, aadA, dfrA, cmlA, cat1, cat2, ereA, aacC4*	*sul1, sul2, sul3, strA, strB, aphA1, tetA, tetB, tetC*	*bla*_TEM_, *bla*_CTX-M_, *bla*_CMY_	Yes	RAPD	ST100	Replicon	Yes	Chapman et al., 2006; Wu et al., 2007; Smith et al., 2010; Abraham et al., 2012, 2014, 2015
					Serogrouping	ST29			
					Phylotyping	ST90			
						ST746			
Austria			*bla*_CTX-M_, *bla*_TEM_, *bla*_SHV_	Yes			Replicon		Mayrhofer et al., 2004, 2006; Petternel et al., 2014
Brazil				Yes	ERIC-PCR			Yes	Carvalho et al., 1991; da Costa et al., 2008; Borges et al., 2012; Morales et al., 2012
					Serogrouping				
Canada	*aadA, dfrA, aadB, aacC4, cmlA*	*sul1, sul2, sul3, strA, strB, catA1, aphA, floR, tetA, tetB, tetC*	*bla*_TEM_, *bla*_OXA_, *bla*_CMY_	Yes	ERIC-PCR				Maynard et al., 2003; Lu et al., 2005; Travis et al., 2006; Duriez and Topp, 2007; Kozak et al., 2009a,b; Rosengren et al., 2009; Deckert et al., 2010; Sheikh et al., 2012
					BOX-PCR				
					Serogrouping				
China	*intI1, aadA, dfrA*	*sul1, sul2, oqxAB*, *aac(6)-lb-cr*, *qnrA, qnrB, qnrS, qepA, fosA, floR*	*bla*_CTX-M_, *bla*_TEM_, *bla*_SHV_, *bla*_CMY_	Yes	Serogrouping	ST10	Replicon	Yes	Yang et al., 2004, 2014; Yue et al., 2008; Ho et al., 2009, 2013; Ma et al., 2009; Huang et al., 2012; Tian et al., 2012; Liu et al., 2013; Meng et al., 2014; Xu et al., 2014; Gao et al., 2015; Liao et al., 2015
					XbaI PFGE	ST206			
					ERIC-PCR				
					Phylogrouping				
Denmark	*intI1, aacC4*	*sul1, sul2, sul3*	*bla*_CTX-M_, *bla*_TEM_, *bla*_CMY_, *bla*_SHV_	Yes	Xbal PFGE	ST10	Replicon	Yes	Olesen et al., 2004; Hammerum et al., 2006, 2014; Jensen et al., 2006; Cavaco et al., 2008; Trobos et al., 2009; Jakobsen et al., 2010a,b; Wu et al., 2010; Agerso et al., 2012; Agerso and Aarestrup, 2013; Hansen et al., 2013; Herrero-Fresno et al., 2015
					Phylogrouping	ST23			
					Serogrouping				
Germany	*intI1, intI2, aadA, dfrA, aadB*	*sul1, sul2, sul3, strA, strB, tetA, tetB, tetM*	*bla*_TEM_, *bla*_CTX-M_, *bla*_OXA_	Yes	Xbal PFGE	ST10	Replicon	Yes	Kadlec and Schwarz, 2008; Schwaiger et al., 2010; Bednorz et al., 2013; Schierack et al., 2013; Fischer et al., 2014; Dahms et al., 2015; Garcia-Cobos et al., 2015
					Phylogrouping	ST58	pMLST		
					Serotyping	ST167			
						ST410			
India			*bla*_CTX-M_*, bla*_SHV_, *bla*_TEM_	Yes	Serogrouping			Yes	Murugan et al., 2012; Rajkhowa and Sarma, 2014; Samanta et al., 2015
Italy			*bla*_CTX-M_, *bla*_TEM_	Yes					Stefani et al., 2014; Luppi et al., 2015
Japan	*intI1, aadA, dfrA, cmlA, aacA4*	*cat1, tetA, tetB, tetC*		Yes	PFGE			Yes	Kijima-Tanaka et al., 2003, 2005; Asai et al., 2005; Harada et al., 2005, 2006; Kumai et al., 2005; Katsunuma et al., 2008; Kojima et al., 2009; Makita et al., 2016
					Phylogrouping				
Korea	*intI1, aadA, dfrA, aadB, cmlA*	*aphA, qnrB, qnrS, aac(6)-lb-cr, tetA*	*bla*_TEM_, *bla*_SHV_, *bla*_CTX-M_	Yes	PFGE			Yes	Choi et al., 2002; Han et al., 2002; Kang et al., 2005; Lim et al., 2007, 2009; Rayamajhi et al., 2008; Unno et al., 2010, 2011; Tamang et al., 2012; Lee et al., 2014
					HFERP				
					Phylogrouping				
					Serogrouping				
Lithuania	*intI1, intI2, aadA, dfrA, estX, sat2*			Yes					Povilonis et al., 2010; Seputiene et al., 2010
Nigeria		*qnrS*	*bla*_TEM_	Yes	ERIC-PCR		pMLST	Yes	Ojo et al., 2010; Fortini et al., 2011; Adenipekun et al., 2015
					PFGE				
					Serogrouping				
Norway	*intI1, intI2, aadA, dfrA, catB, sat1*	*sul1, sul2, strA, strB, tetA, tetB, tetC*	*bla*_TEM_, *bla*_OXA_	Yes	PFGE	ST10			Sunde et al., 1998; Sunde and Sorum, 1999; Brun et al., 2002; Sunde, 2005; Sunde and Norstrom, 2006; Trobos et al., 2009
						ST168			
Portugal	*intI1, intI2, dfrA, aadA, estX, sat2, catB*	*strA, strB, qnrB, tetA*	*bla*_TEM_, *bla*_CTX-M_, *bla*_SHV_	Yes	XbaI PFGE	ST10	Replicon		Machado et al., 2008; Goncalves et al., 2010; Jones-Dias et al., 2013; Ramos et al., 2013; Rodrigues et al., 2013
							pMLST		
							RFLP		
Spain	*intI1, intI2, aadA, dfrA, cmlA, sat2, linF*	*sul1, sul3, pmrA, pmrB*	*bla*_CTX-M_, *bla*_TEM_, *bla*_SHV,_ *bla*_OXA_	Yes	Phylogrouping				Brinas et al., 2002; Sabate et al., 2008; Escudero et al., 2010; Marchant and Moreno, 2013; Marchant et al., 2013; Ojer-Usoz et al., 2013; Quesada et al., 2015
					REP-PCR				
					ERIC-PCR				
Switzerland	*intI1, aadA*	*sul1, sul2, sul3, strA, strB, tetA, tetB, tetC*	*bla*_CTX-M_	Yes	PFGE	ST3		Yes	Stephan and Schumacher, 2001; Lanz et al., 2003; Perreten and Boerlin, 2003; Zweifel et al., 2006; Stannarius et al., 2009; Endimiani et al., 2012
					Serogrouping	ST529			
Taiwan	*intI1, aadA, dfrA*	*sul1, fosA*	*bla*_TEM_, *bla*_CTX-M_, *bla*_CMY_	Yes	XbaI PFGE	ST744			Hsu et al., 2006; Tseng et al., 2015
						ST2310			
Thailand	*intI1, aadA, dfrA, linF, sat1*	*sul1*	*bla*_CTX-M_, *bla*_TEM_	Yes	Phylogrouping			Yes	Hanson et al., 2002; Phongpaichit et al., 2007; Mathew et al., 2009; Lay et al., 2012; Changkaew et al., 2015
UK	*aadA*	*sul2, floR*	*bla*_CTX-M_, *bla*_SHV_	Yes			pMLST		Jackson, 1981; Enne et al., 2008; Taylor et al., 2008; Freire Martin et al., 2014; Randall et al., 2014; Cheney et al., 2015
USA	*intI1, aadA, dfrA, cmlA, sat1*	*cat2, flo, tetA, tetB, tetC, tetM*		Yes	Serogrouping		Replicon	Yes	Bischoff et al., 2002; Schroeder et al., 2002; Bryan et al., 2004; Singh et al., 2005; Lindsey et al., 2011; Xia et al., 2011; Ju et al., 2012; Tadesse et al., 2012; Roug et al., 2013
					XbaI PFGE				
					Phylogrouping				


Much needs to be done to reduce the reliance on antimicrobials in food production. There is a growing body of evidence suggesting that food production may be a key driver in the evolution of multiple drug resistance in a number of bacterial species of clinical significance to human health. Livestock represent a reservoir of *E. coli* pathotypes with resistance to more than one antimicrobial. While pigs and poultry are significant sources of ExPEC, cattle are the major reservoirs for Shiga toxin producing *E. coli* (EHEC; Manges and Johnson, 2015) and atypical EPEC (Hornitzky et al., 2005; Dawes et al., 2010). The consequences for the environment of large-scale intensive animal production and a growing human population, estimated to be 9 billion by 2050 (United Nations, 2015) are not fully understood. Much remains to be done to determine how antimicrobial resistance genes are assembled on mobile genetic elements and to identify their unique genetic features. Diagnostics tests that target unique features will be useful in tracking the movement of mobile elements that are carrying complex resistance loci and to identify hotspots where they reside. Metagenomic approaches will have a major role to play in understanding the scale of the antimicrobial resistance problem, detecting and tracking emerging pathogens, and the role of gut flora in human and animal health. Studies on microbial populations from the gastrointestinal tracts of intensively reared livestock are needed to identify and characterize novel pathotypes that will undoubtedly emerge in response to the selection pressures that are commonplace in large-scale animal production facilities. Genomic surveillance studies will have an important role to play in identifying complex resistance loci, particularly how they move through different production systems and natural ecosystems (aquatic and terrestrial), and the impact they have on the carriage of multiple antimicrobial resistance genes in bacteria that colonize and infect humans and animals. Currently, there are just three complete porcine-derived *E. coli* chromosomal sequences published, as well as a variety of complete plasmid sequences, the majority of which have been derived from the study of isolates with closed genome sequences (**Table 2**). This is at odds with the significant role that intensive pig production plays in the release of antibiotic residues into the environment, and we expect more focus to be placed on the sequencing of pig-associated *E. coli* isolates in the near future.

**Table 2:** Published complete porcine *E. coli* genome and plasmid sequences.

Name	Country	Type	Size (bp)	Achtman MLST/Plasmid Inc.	Disease association/source	GenBank accession	Reference
pND11_107	USA	Plasmid	107,138	I	Neonatal diarrhea	HQ114281	Johnson et al., 2011
pPWD4_103	USA	Plasmid	103,297	I	Post-weaning diarrhea	HQ114284	
pUMNF18_69	USA	Plasmid	69,065	I		CP002891	
pND12_96	USA	Plasmid	92,290	I	Neonatal diarrhea	HQ114282	
UMNK88	USA	Chromosome	5,186,416	ST165 cplx	Post-weaning diarrhea	CP002729	Shepard et al., 2012
pUMNK88_Hly	USA	Plasmid	65,549			CP002733	
pUMNK88_Ent	USA	Plasmid	81,475			CP002732	
pUMNK88_K88	USA	Plasmid	81,883			CP002730	
pUMNK88_91	USA	Plasmid	90,868	I		CP002731	
pUMNK88	USA	Plasmid	160,573	A/C		HQ023862	
pTC1	Hungary	Plasmid	91,019		Weaning diarrhea	CP000913	Fekete et al., 2012
pHK23a	China	Plasmid	73,607	FII	Multi-drug resistant isolate	JQ432559	Ho et al., 2013
pSCEC2	China	Plasmid	135,615	A/C	Maxillary lymph node	KF152885	Zhang et al., 2014
pIFM3804	UK	Plasmid	104,399	I		KF787110	Freire Martin et al., 2014
PCN033	China	Chromosome	4,987,957	ST5147	Extraintestinal infection (brain)	CP006632	Liu et al., 2015
PCN033p1	China	Plasmid	3,319			CP006633	
PCN033p2	China	Plasmid	4,086			CP006634	
PCN033p3	China	Plasmid	161,511			CP006635	
PCN061	China	Chromosome	4,603,777	ST46	Extraintestinal infection (lung)	CP006636	
PCN061p1	China	Plasmid	2,014			CP006637	
PCN061p2	China	Plasmid	5,754			CP006638	
PCN061p3	China	Plasmid	6,222	Q1/FIB/FIC/FII		CP006639	
PCN061p4	China	Plasmid	34,692			CP006640	
PCN061p5	China	Plasmid	103,644	I		CP006641	
PCN061p6	China	Plasmid	145,722	N/FIB/X1		CP006642	
pFSEC-01	China	Plasmid	33,885			KR779901	Zhang et al., 2015
pSD11	China	Plasmid	37,672	X4		KM212169	Sun et al., 2015


## Author Contributions

EW drafted an early version of the manuscript and produced the figure and tables. PR and IC contributed to several sections in the manuscript. TC and JH edited drafts of the manuscript. SD wrote most the manuscript.

## Conflict of Interest Statement

The authors declare that the research was conducted in the absence of any commercial or financial relationships that could be construed as a potential conflict of interest.
